# Effects of Diets Containing Different Levels of Copper, Manganese, and Iodine on Rumen Fermentation, Blood Parameters, and Growth Performance of Yaks

**DOI:** 10.3390/ani13162651

**Published:** 2023-08-17

**Authors:** Huizhen Lu, Weibin Wu, Xinsheng Zhao, Musaddiq Wada Abbas, Shujie Liu, Lizhuang Hao, Yanfeng Xue

**Affiliations:** 1 College of Animal Science and Technology, Anhui Agricultural University, Hefei 230036, China; luhuizhen1992@163.com (H.L.); wuweibin2000@foxmail.com (W.W.); musaddiqabbas@gmail.com (M.W.A.); 2 Biotechnology Centre, Anhui Agricultural University, Hefei 230036, China; 3 State Key Laboratory of Plateau Ecology and Agriculture, Key Laboratory of Plateau Grazing Animal Nutrition and Feed Science of Qinghai Province, Qinghai Plateau Yak Research Center, Qinghai Academy of Science and Veterinary Medicine, Qinghai University, Xining 810016, China; zhaoxinsheng1997@126.com (X.Z.); mkylshj@126.com (S.L.)

**Keywords:** yak, trace element, growth performance, rumen fermentation, blood index

## Abstract

**Simple Summary:**

The yak is the primary income source for millions of people in remote areas of western China and other parts of Asia, providing a basic subsistence commodity for herdsmen. Yaks depend on natural grazing, but the grass trace elements may be insufficient for the yaks’ requirements. Our prior research demonstrated that the levels and sources of copper, manganese, and iodine could influence in vitro rumen fermentation for yaks. The aim of this study was to optimize yak feeding standards by examining the impact of diets containing different levels of copper, manganese, and iodine on yak growth. Results showed that diets containing 15.00 mg/kg of copper, 50.00 mg/kg of manganese, and 0.50 mg/kg of iodine had the most positive effect on the growth of growing yaks. Our study will facilitate yak diet formulation design and promote yaks’ industry development.

**Abstract:**

Copper, manganese, and iodine are part of a yak’s required trace elements. However, knowledge about their dietary requirements is scarce. Therefore, an experiment was conducted to evaluate rumen fermentation, blood parameters, and growth performance and screen out the optimum levels of trace elements in yaks’ diet. Here, 18 three-year-old castrated yaks were randomly divided into four groups, which fed with diets containing basal (CON: 4.40, 33.82, and 0 mg/kg) and low-level (LL: 10.00, 40.00, and 0.30 mg/kg), middle-level (ML: 15.00, 50.00, and 0.50 mg/kg), and high-level (HL: 20.00, 60.00, and 0.70 mg/kg) copper, manganese, and iodine for 30 days. With the increase in trace elements, yaks’ daily weight gain (DWG), rumen pH, ammonia nitrogen, microbial protein (MCP), and volatile fatty acids levels and serum triglycerides and urea nitrogen levels showed firstly increasing and then decreasing trends and reached the highest values in ML, and serum ceruloplasmin and total superoxide dismutase (T-SOD) activities showed continuously increasing trends. Yaks’ DWG, rumen MCP, butyrate, and valerate levels and serum triglycerides, urea nitrogen, ceruloplasmin, and T-SOD levels in ML were significantly higher than CON. Therefore, the recommended levels of copper, manganese, and iodine in growing yaks’ diet are 15.00, 50.00, and 0.50 mg/kg (ML), respectively.

## 1. Introduction

The yak is one of the world’s most extraordinary bovines, which has evolved to survive under high altitude, cold weather, and low-oxygen-concentration conditions [1]. Although China has the most yaks in the world, yaks can also be found in Afghanistan, Pakistan, India, Mongolia, and Russia [1]. The yak is the main income source for millions of people in remote areas of western China and other parts of Asia. They provide fundamental subsistence commodities such as meat, hides, milk, butter, rope, fuel, and felt for herdsmen [2]. Yaks depend on natural grazing, but the grass trace elements may be not sufficient for the yak’s requirements [3]. In addition, due to the harsh living conditions and forage fluctuation, yaks are strong in the summer, fat in the autumn, thin in the winter, and weak or even dead in the spring [4]. In order to avoid yak weight loss and reduce yaks’ mortality in the cold season, diet supplementation is important. However, because there is little knowledge about the nutritional requirements of yaks, there is a lack of a good feeding standard to make a supplemental diet. Previously, various studies on yak metabolic energy [5] and crude protein [6] requirements were conducted. However, feeding guidelines for yak trace element requirements are inadequately documented.

Although trace elements take up only a small proportion of the overall body weight, they play important roles in many biochemical and physiological processes related to health, growth, lactation, and reproduction, such as oxygen transfer, hormone production, enzyme activity, vitamin synthesis, collagen formation, tissue synthesis, and chemical energy production [7]. Copper, manganese, iodine, iron, zinc, selenium, molybdenum, and cobalt are all essential trace elements for animals. Copper plays a crucial role in biological redox processes by regulating the activity of copper-containing oxidases [8]. Copper supplementation is necessary since grazing yaks are prone to copper deficiency disease, commonly known as “swayback disease”, with an estimated prevalence of 50% to 60% and mortality rates of up to 70% [9]. Manganese is required for the enzymes glycosyltransferase and galactotransferase, which are required for appropriate cartilage and skeletal formation and progression [10]. Manganese strongly influences skeletal development, growth, reproduction, immunity, and neurological and emotional activity [11]. Iodine is involved in the synthesis of thyroxine; regulates the metabolism of minerals, sugars, fats, proteins, and vitamins; enhances enzyme activity; and promotes the body’s growth and development [12]. Iodine deficiency can cause thyroid gland enlargement and indirectly influence the growth rate. Despite the importance of trace elements such as copper, manganese, and iodine, there is limited international research on these elements in yaks, which causes the inadequate supplementation or over-supplementation of trace elements in yaks’ diet. However, both trace element deficiency and over-supplementation will affect yaks’ health and productivity. Previously, we used in vitro yak rumen fermentation to explore the optimal sources and level ranges of copper, manganese, and iodine for yaks and found that copper-methionine, manganese-methionine, and Ca(IO_3_)_2_ were the optimal sources of copper, manganese, and iodine and that 10.00–20.00 mg/kg of copper, 40.00–60.00 mg/kg of manganese, and 0.30–0.70 mg/kg of iodine were the optimal level ranges in yaks’ diet [13,14,15]. Further, copper showed antagonism with molybdenum, zinc, sulfur, silver, cadmium, calcium, and phosphorus and showed synergistic effects with iron and cobalt. Manganese showed antagonism with phosphorus, calcium, magnesium, natrium, and iron and showed synergistic effects with molybdenum and cobalt. Iodine showed antagonism with arsenic, fluorine, cobalt, chlorine, molybdenum, calcium, and mercury and showed synergistic effects with phosphorus [16,17]. The interactions among copper, manganese, and iodine are relatively weak, and the optimal levels of copper, manganese, and iodine for growing yaks can be detected simultaneously in vivo.

Mineral nutrition research is increasing recently, and many feed additives have been created and promoted. Mineral supplementation of animal diets has become a vital production measure, and the amount of mineral supplementation is growing. However, over-supplementation with trace minerals can lead to a reduction in animal performance [18]. Here, it was hypothesized that a yak diet with optimal levels of trace minerals would improve yaks’ rumen fermentation and body metabolism to finally elevate yaks’ growth performance. The objective of this study was to screen out the optimum levels of copper, manganese, and iodine in the yak diet by investigating the effects of diets containing different levels of copper, manganese, and iodine on yaks’ growth performance, rumen fermentation, and metabolic parameters, which would finally contribute to optimizing yaks’ feeding standards.

## 2. Materials and Methods

### 2.1. Experimental Location and Animal Diet Design

All animal care and treatment procedures were approved by the Animal Use Committee of the Academy of Science and Veterinary Medicine of Qinghai University (Approval No. QHU20150301).

The experiment was conducted at the Yak Demonstration Park (Longitude E 100.95°, Latitude N 36.92°, Attitude 3221 m) on the Qinghai–Tibet Plateau. The energy and protein contents in the diet were designed based on the requirement of a 150.00 kg yak with 500.00 g daily weight gain (DWG) according to the China Standard of Beef Cattle (NY/T 815-2004) [19] and Yak Nutrition Monograph [20]. The diet was prepared in a 6:4 concentrate to roughage.

### 2.2. Animal Experimental Design and Daily Weight Gain

The experiment was conducted on 18 three-year-old castrated yaks with similar body weights (152.97 ± 6.98 kg), which were allocated into three treatment groups with five replicates each and a control group (CON) with three replicates. Yaks were fed twice a day at 8:00 and 18:00 (4.00 kg/day) in individual pens and had free access to water. The optimal sources and level ranges of copper, manganese, and iodine were initially determined through in vitro yak rumen fermentation [13,14,15]. Due to the extremely high contents of copper, manganese, and iodine in the trace elements products, the supplementary amount of the products in the yaks’ diet was extremely low. It would be very difficult to precisely control the supplementary amount of the trace elements products. Therefore, the concentrate diet and coarse oat green hay were first physically crushed, passed through a forty-mesh sieve (aperture 0.45 mm), and then mixed thoroughly to form the basal diet according to a 6:4 ratio of concentrate to coarse. The copper-methionine, manganese-methionine, and Ca(IO_3_)_2_ products were then diluted with a small portion of the basal diet, and the diluted diet was then added to the full basal diet to create a concentration gradient of copper, manganese, and iodine in the diets of the different treatment groups. To form the low-level group (LL) yak diet, the basal diet was supplemented with 46.71 mg/kg of copper-methionine, 51.51 mg/kg of manganese-methionine, and 3.00 mg/kg of Ca(IO_3_)_2_. The middle-level group (ML) yak diet was formed by adding 88.38 mg/kg of copper-methionine, 134.84 mg/kg of manganese-methionine, and 5.00 mg/kg of Ca(IO_3_)_2_ to the basal diet. The high-level group (HL) yak diet was formed by supplementing the basal diet with 130.04 mg/kg of copper-methionine, 218.18 mg/kg of manganese-methionine, and 7.00 mg/kg of Ca(IO_3_)_2_. Finally, the diet (basal diet) of yaks in CON contains 4.40 mg/kg of copper, 33.82 mg/kg of manganese, and 0 mg/kg of iodine (undetected). The diet of yaks in LL contains a total of 10.00 mg/kg of copper, 40.00 mg/kg of manganese, and 0.30 mg/kg of iodine. The diet of yaks in ML contains a total of 15.00 mg/kg of copper, 50.00 mg/kg of manganese, and 0.50 mg/kg of iodine. The diet of yaks in HL contains a total of 20.00 mg/kg of copper, 60.00 mg/kg of manganese, and 0.70 mg/kg of iodine. The detailed ingredients and nutritional levels of the diets for yaks in different groups are shown in Table 1. The yaks’ body weights were measured prior to morning feeding before and after the 30-day treatment to calculate DWG.

### 2.3. Determination of Trace Element Content in the Basal Diet

Five aliquot samples were randomly collected from the total mixed basal diet to detect the concentrations of trace elements. The determination of copper, manganese, and iodine levels in all of the basal diet samples was conducted in triplicate to ensure the accuracy and reliability of the results. The concentrations of copper and manganese in the basal diet were determined according to the national standard (GB/T 13885-2003) [21]. Firstly, a 5.00 mg basal diet sample was carbonized in a crucible and transferred to a muffle furnace (SX2-4-10A, Shanghai Jiecheng Experimental Instrument Co., Ltd., Shanghai, China) for 4 h at 550 ± 15 °C for ash. After cooling to room temperature, the ash was thoroughly mixed with 5 mL of 6 mol/L hydrochloric acid. When the ashes were completely dissolved, they were transferred to a volumetric flask and diluted to 50.00 mL. The contents of trace elements copper and manganese in the solution were determined using an atomic absorption spectrophotometer (AAS6000, Jiangsu Skyray Instrument Co., Kunshan, China). The parameters of the atomic absorption spectrophotometer for determining trace elements copper and manganese in the diets were set at wavelengths 324.80 nm and 279.50 nm, slit widths 0.70 nm and 0.20 nm, light mode NON-BGC, and minimum lamp currents 6.00 mA and 10.00 mA, respectively. The iodine content in the basal diet was measured by the method described by Christian et al. [22].

### 2.4. Blood Samples Collection and Blood Indicators Detection

Blood samples were collected from the jugular vein of yaks prior to morning feeding before and after the 30-day treatment. The collected samples were then centrifuged at 1200× *g* for 15 min to obtain serum for further analysis. The serum was entrusted to the Qinghai Provincial People’s Hospital to determine routine enzyme activity including glutathione aminotransferase (ALT), glutathione transaminase (AST), alkaline phosphatase (ALP), and lactate dehydrogenase (LDH); protein indicators including total protein, albumin, globulin, and blood urea nitrogen contents; energy indicators including total cholesterol, triglyceride, and glucose contents; and routine mineral contents including potassium, sodium, chlorine, calcium, phosphorus, magnesium, and iron using an automatic blood biochemistry analyzer (BK-600, BIOBASE, Jinan, China). Serum ceruloplasmin (CLP) activity and total superoxide dismutase (T-SOD) activity, which are highly correlated with trace elements copper and manganese, were determined using Nanjing Jiancheng Biological Institute kits based on the manufacturers’ handbooks.

### 2.5. Ruminal Fluid Samples Collection and Fermentation Parameters Detection

Rumen fluid samples (about 200.00 mL) were sucked from the rumen through the oral cavity of yaks using a rumen fluid collector prior to morning feeding after a 30-day treatment. The pH of rumen fluid was immediately measured using a high-precision acidity meter (HI221, HANNA, Padua, Italy). The fluid was then filtered through four layers of gauze to remove impurities and stored in liquid nitrogen for the detection of yak rumen fermentation parameters. The concentration of ammonia nitrogen (NH_3_-N) was determined using a spectrophotometer (UV5, METTLER TOLEDO Technology Co., Shanghai, China) following the method described by Feng et al. [23]. The Comas Brilliant Blue method was utilized to measure the microbial protein (MCP) content of the rumen fluid samples [24]. The volatile fatty acids (VFAs) contents in the rumen fluid were measured using gas chromatography equipped with a capillary column of 30.00 m × 0.32 mm × 0.50 µm. The detector’s hydrogen pressure and air pressure were set to 0.40 MPa, while the capillary column pressure was set to 0.60–0.80 MPa [24].

### 2.6. Statistical Analysis

Data from the experiment were analyzed using an unbalanced completely randomized design with the Statistical Analysis System (SAS) version 9.0 software. Comparisons were conducted using one-way ANOVA and Duncan’s method. Polynomial comparisons (unequal spacing) were used to determine the linear and quadratic effects of mineral supplementation levels. Results are presented as mean ± standard deviation (SD). *p* ≤ 0.05 was used to identify the significant differences among different groups, and *p* < 0.1 was used to identify the linear or quadratic changing trends for the detected indicators with the increasing trace element levels.

## 3. Results

### 3.1. Effect of Trace Elements on Yaks’ Growth Performance and Average Daily Feed Intake

**Figure 1:**
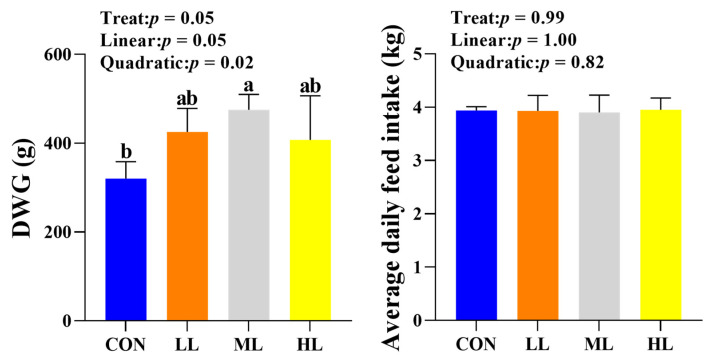
Effect of different trace element levels on growth performance and average daily feed intake of yaks. Different letter superscripts indicate significant difference (*p* < 0.05), while same letter superscripts indicate no significant difference (*p* > 0.05). DWG: Daily weight gain; CON: *n* = 3; LL, ML, and HL: *n* = 5.

With the increasing trace element levels, the DWG of yaks exhibited a quadratic change (*p* = 0.02), which increased first and then decreased and reached a peak value in ML. The overall difference in DWG among the four groups was significant (*p* = 0.05). Moreover, the DWGs of yaks in LL, ML, and HL were higher than that of CON, and only the difference in the DWG between ML and CON was found to be significant (*p* < 0.05). The average daily feed intake was not found to be significantly different between any two groups (*p* > 0.05) (Figure 1).

### 3.2. Effect of Trace Elements on Yak’s Rumen Fermentation

Rumen fermentation is important for the growth performance of ruminants. In order to dissect the potential mechanism of different DWGs among groups, rumen pH, NH_3_-N, and MCP levels were detected. Overall, pH (*p* = 0.04), NH_3_-N (*p* = 0.06), and MCP levels (*p* < 0.01) in yak rumen exhibited a quadratic change with increasing trace element levels. Specifically, pH, NH_3_-N, and MCP levels increased first and then decreased, all reaching the highest values in ML. The overall difference in ruminal MCP levels among the four groups was significant (*p* < 0.01). The ruminal MCP level of ML reached the highest value of 4.973 g/L, which was markedly higher than those of LL and CON (*p* < 0.05) but was not markedly different from that of HL (*p* > 0.05) (Table 2).

Propionate (*p* = 0.08), Isobutyrate (*p* = 0.06), Butyrate (*p* = 0.04), Isovalerate (*p* = 0.07), Valerate (*p* = 0.02), and total VFAs (*p* = 0.05) levels in yak rumen exhibited a linear change with increasing trace element levels, while acetate (*p* = 0.06) showed a quadratic change. All VFAs increased first and then decreased, with peak values observed in ML. The overall difference in butyrate (*p* = 0.03) and valerate (*p* = 0.05) levels among the four groups was significant. The levels of butyrate and valerate in ML were all higher than the levels in CON (*p* < 0.05). The levels of valerate in HL were also significantly higher (*p* < 0.05) than those in CON. In this study, the ratio of acetate to propionate reached the lowest value in ML, but no significant difference (*p* > 0.05) was identified between any two groups (Table 3).

### 3.3. Effect of Trace Elements on Yak’s Serum Energy, Protein, and Mineral Indicators

Following treatment, the triglyceride (*p* < 0.01) level in yak rumen exhibited a quadratic change with increasing trace element levels, which increased first and then decreased and reached a peak value in ML. After the treatment, the overall difference in triglyceride level (*p* < 0.01) among the four groups was significant. For the comparison between before and after the treatment, triglyceride level in ML after the treatment was markedly (*p* < 0.05) higher than that before the treatment (Table 4).

Following treatment, total protein (*p* = 0.08) and blood urea nitrogen (*p* < 0.01) levels in yak serum exhibited a quadratic change with increasing trace element levels. Blood urea nitrogen increased first and then decreased, with peak values observed in ML. After the treatment, the overall difference in blood urea nitrogen level (*p* = 0.01) among the four groups was significant. For the comparison between before and after the treatment, only blood urea nitrogen levels in HL and CON after the treatment were markedly (*p* < 0.05) below those before the treatment (Table 5).

Following treatment, calcium (*p* = 0.04) levels in yak serum exhibited a linear change with increasing trace element levels, while iron (*p* = 0.05) showed a quadratic change. Further, the differences in all of the minerals before and after the treatment in any groups were not significant (*p* > 0.05) (Table 6).

### 3.4. Effect of Trace Elements on Yak Serum Enzyme Activities

Following treatment, CLP (*p* < 0.01) and T-SOD (*p* < 0.01) activities in yak serum exhibited a linear increasing change with increasing trace element levels. After the treatment, the overall difference in CLP (*p* < 0.01) and T-SOD activities (*p* < 0.01) among the four groups was significant. Moreover, both CLP and T-SOD activities of ML and HL were significantly higher than those of LL and CON (*p* < 0.05), and both CLP and T-SOD activities of LL were markedly higher than those of CON (*p* < 0.05), while no differences (*p* > 0.05) were found for CLP and T-SOD activities between ML and HL (Table 7).

### 3.5. Comparison of Yaks’ Trace Elements Requirements with Those of Cattle in Different Nutritional Standards

This research found that diets containing 15.00 mg/kg of copper, 50.00 mg/kg of manganese, and 0.50 mg/kg of iodine had the most positive effect on the growth of growing yaks. According to the NRC (1989) [25], NRC (2000) [25], ARC (1980) [25], NY/T (2004) [19], CSIRO (2007) [26], NorFor (2011) [27], and Tolerance level [25] nutrition standards, the requirements of copper, manganese, and iodine for growing cattle were 10.00–15.00 mg/kg, 20.00–40.00 mg/kg, and 0.25–0.50 mg/kg (Table 8).

## 4. Discussion

Although the energy and protein content of a diet is widely considered to be the most important factor affecting livestock production, minerals can also affect animal growth, reproductive performance, and immune function [28]. Growing sheep that were fed with diets supplementing trace elements containing copper (21.80 mg/kg) and manganese (48.00 mg/kg) showed improved growth performance [29]. However, it was observed that feeding diets with high levels of copper (25.00 mg/kg) could cause toxicity in sheep and affect their growth performance [30]. Our study also found that with increasing levels of copper, manganese, and iodine, yaks’ growth performance increased first and then decreased, which reached a peak in ML. Therefore, trace elements supplementation could improve yaks’ growth performance, and the levels of copper, manganese, and iodine in ML are the optimal choice for growing yaks in terms of growth performance.

The pH is an important indicator of a stable internal environment for rumen, which can influence rumen microbial activity and fermentation parameters [31]. In this study, yaks’ rumen pH ranges from 6.91 to 7.05 within the normal range of 6.2 to 7.2 [32] and indicates that the additive levels of trace elements are responsible for the stabilization of rumen pH. Ruminal NH_3_-N is generated by breaking down protein or non-protein nitrogen present in the feed, which is further employed as a fundamental material by microorganisms for the synthesis of MCP [33]. Hence, adequate NH_3_-N concentration is a key factor in ensuring effective MCP synthesis. MCP synthesized in the rumen is an important source of essential amino acids for ruminants and plays an essential role in their nutrition. The in vitro yak rumen fermentation assay had shown that the levels of NH_3_-N and MCP increase first and then decrease with increasing copper levels, and both peaked at 10.00–15.00 mg/kg of copper in the diet [13]. In the present study, ruminal NH_3_-N and MCP levels increased and then decreased with the increase in trace elements, reaching the highest values in the ML with 15.00 mg/kg of copper, 50.00 mg/kg of manganese, and 0.50 mg/kg of I in the diet, which are highly consistent with the references [13]. VFAs, which are produced by the microbial breakdown of carbohydrates in feed, are the primary source of energy for the growth and reproduction of rumen microbes as well as the host ruminant [34]. Earlier studies had suggested that feeding Elvet goats with a diet containing 17.46 mg/kg of copper [35] and cattle with a diet containing 62.33 mg/kg of manganese [36] led to a significant increase in the total concentration of VFAs in the rumen. However, Angus cattle fed too high levels of copper (57.30 mg/kg) showed significantly decreased concentrations of acetate, propionate, butyrate, and total VFAs in the rumen [37]. Calves fed a diet containing too high concentrations of manganese (3000.00 mg/kg) diet also showed significantly reduced concentrations of propionate and total VFAs in the rumen [38]. Supplementation with a low concentration of iodine (10.00 mg/kg) had no effect on the concentrations of VFAs in the rumen of calves [39]. The current study also found that VFAs concentrations in yak rumen showed firstly increasing and then decreasing trends with the increasing levels of trace elements and reached maximum values in ML. The addition of appropriate levels of trace elements probably increased rumen microbial activities and digestive enzymes activities, which promoted the degradation and digestion of feed and increased rumen fermentation parameters including the levels of NH_3_-N, MCP, and VFAs in the rumen, and this finally improved yak’s growth performance.

Blood metabolites are generally useful indicators of animal health and often reflect the metabolic homeostasis of the body. Levels of triglyceride and total cholesterol in the blood are commonly used to evaluate energy metabolism in dairy cows and small ruminants because they are closely linked to blood glucose concentration [40]. Blood cholesterol levels were significantly increased when ewes were injected with trace elements containing copper, manganese, and iodine [41]. It had been observed that blood triglyceride levels decreased when Najdi ewes were supplemented with too high levels of trace elements containing copper (3.94 mg), manganese (3013.00 mg), and iodine (330.00 mg) [42]. In the present experiment, serum cholesterol and triglyceride levels in yaks showed an increasing and then decreasing trend with increasing trace element levels, and both reached the maximum values in ML, which complies with the references. Apart from the energetic and nutritional contributions, VFAs can indirectly affect the synthesis of cholesterol [43] and triglyceride [44]. Therefore, the changing trend of blood triglyceride and cholesterol might result from the variation in VFAs concentration in yak rumen in response to different levels of trace elements in their diet. Total protein, albumin, globulin, blood urea nitrogen, and albumin/globulin levels reflect the organism’s digestive metabolism and nitrogen utilization. Blood urea nitrogen level is the dynamic result of ammonia absorption and urea production and excretion, which relates the metabolism of proteins and amino acids in the organism to the microbiota in the rumen [45,46]. Studies have shown that there is a strong correlation between blood urea nitrogen levels and the levels of NH_3_-N and MCP in the rumen [45]. The synthesis of NH_3_-N and MCP in the rumen of swamp buffalo has been found to be strongly linked to blood urea nitrogen concentration according to various studies [47,48,49]. In this study, yaks’ blood urea nitrogen level increased first and then decreased with the increase in trace elements, which is also similar to the variation trend of NH_3_-N and MCP levels in the rumen. Thus, the levels of copper, manganese, and iodine in ML are the optimal levels for yaks’ nitrogen utilization. CLP is a copper-containing enzyme protein and is the most sensitive indicator of copper nutrition in animals. It has both antioxidative and oxidative activity and can catalyze the oxidation of polyphenols and polyamine substrates [50]. Copper, manganese, and zinc are structural and functional components of T-SOD, which is a major antioxidant with a strong free radical scavenging capacity [51,52]. There was evidence that blood CLP and T-SOD activities were significantly increased in Tibetan sheep supplemented with mineral blocks for a long period [53] and in piglets fed a copper-containing diet [54]. In the current study, yak’s blood CLP and T-SOD activities showed a continuously increasing trend with the increase in copper, manganese, and iodine levels in the diet.

Synergy and antagonism are widely observed between different minerals [55]. We were curious whether our supplementation of copper, manganese, and iodine affects the levels of other macroelements and microelements, so some mineral indicators in yak serum were tested. Our results show that adding levels of trace elements in the current study does not affect the metabolism of other elements including potassium, sodium, chlorine, calcium, phosphorus, magnesium, and iron. ALT and AST are two key hepatic aminotransferases, and blood ALT/AST levels are important indicators of liver function in animals [56]. ALP relates to nucleic acid metabolism and LDH involved in gluconeogenic enzymatic reaction [57], and blood ALP and LDH activities are widely used as indicators of liver diseases [58]. In the present study, none of these above indicators were changed significantly upon copper, manganese, and iodine supplementation, suggesting that increasing trace element levels had no effect on yak liver function.

Taken together, optimal levels of copper, manganese, and iodine (15.00 mg/kg of copper, 50.00 mg/kg of manganese, and 0.50 mg/kg of Iodine) significantly increased rumen fermentation indicators including NH_3_-N, MCP, and VFAs levels; serum energy and protein indicators including triglyceride and urea nitrogen levels; and serum antioxidant enzyme activities including CLP and T-SOD in growing yaks. Therefore, optimal copper, manganese, and iodine levels in the yaks’ diet improved rumen fermentation, body metabolism, and antioxidant capacity, ultimately improving yak growth performance.

As compared to the nutrition standards of growing cattle, the optimal levels of copper (15.00 mg/kg) and iodine (0.50 mg/kg) for growing yaks are at the maximum values of copper (10.00–15.00 mg/kg) and iodine (0.25–0.50 mg/kg) requirements for growing cattle, while the optimal level of manganese (50.00 mg/kg) for growing yaks is slightly higher than the manganese requirement (20.00–40.00 mg/kg) for growing cattle. The relatively higher requirements of copper, manganese, and iodine for growing yaks compared to growing cattle probably result from the different survival environment (high attitude, extremely cold, low oxygen pressure, and low-quality forage) and long-period evolutionary mechanisms [59,60].

## 5. Conclusions

Trace elements supplementation could improve yak rumen fermentation to increase the levels of NH_3_-N, MCP, and VFAs in the rumen and elevate the levels of triglyceride and urea nitrogen and the activities of CLP and T-SOD in the serum. The optimal levels of copper, manganese, and iodine in growing yaks’ (~150 kg BW) diet were 15.00 mg/kg, 50.00 mg/kg, and 0.50 mg/kg, respectively, which improved rumen fermentation, body metabolism, and antioxidant capacity, ultimately improving yak growth performance. This study provided a novel theoretical foundation for the establishment of yaks’ feeding standard.

## Figures and Tables

**Table 1:** The ingredients and nutritional levels of the diets for yaks in different groups ^1^.

Item	CON	Treatments		
		LL	ML	HL
Ingredient composition, (% on DM basis)				
Maize grain	36.59	36.59	36.59	36.59
Wheat bran	12.20	12.20	12.20	12.20
Soybean meal	6.71	6.71	6.71	6.71
Rapeseed meal	2.17	2.17	2.17	2.17
Limestone	0.91	0.91	0.91	0.91
CaHPO_4_	0.17	0.17	0.17	0.17
NaCl	0.59	0.59	0.59	0.59
Oat hay	40.66	40.66	40.66	40.66
Cu-Methionine (mg/kg)	0	46.71	88.38	130.04
Mn-Methionine (mg/kg)	0	51.51	134.84	218.18
Ca(IO_3_)_2_ (mg/kg)	0	3.00	5.00	7.00
Chemical composition, (DM basis) ^2^				
Dry matter (%)	90.98	90.98	90.98	90.98
Total crude protein (%)	11.03	11.03	11.03	11.03
Acid detergent fiber (%)	17.60	17.60	17.60	17.60
Neutral detergent fiber (%)	31.12	31.12	31.12	31.12
Calcium (%)	0.88	0.88	0.88	0.88
Phosphorus (%)	0.19	0.19	0.19	0.19
Copper (mg/kg)	4.40	10.00	15.00	20.00
Manganese (mg/kg)	33.82	40.00	50.00	60.00
Iodine (mg/kg)	0	0.30	0.50	0.70

^1^ CON: Control group; LL: Low-level group; ML: Middle-level group; HL: High-level group; CON: *n* = 3; LL, ML, and HL: *n* = 5. ^2^ The chemical composition values of the diets are analytical values.

**Table 2:** Effect of different trace element levels on pH, NH_3_-N concentration, and MCP in the rumen of growing yaks ^1^.

Item ^2^	CON	LL	ML	HL	*p*-Value ^3^		
					T	L	Q
pH	6.99 ± 0.15	7.01 ± 0.07	7.05 ± 0.05	6.91 ± 0.06	0.06	0.24	0.04
NH_3_-N concentration (mg/dL)	5.38 ± 0.98	6.55 ± 0.63	7.07 ± 0.81	6.36 ± 1.30	0.17	0.14	0.06
MCP content (g/L)	2.31 ± 0.72 ^b^	3.30 ± 0.58 ^ab^	4.97 ± 0.72 ^c^	4.19 ± 0.89 ^ac^	<0.01	<0.01	0.02

^1^ For each superscript, different letters indicate significant difference (*p* < 0.05), while the same letter or no letter indicates no significant difference (*p* > 0.05). Data are shown as mean ± SD. ^2^ NH_3_-N: Ammonia nitrogen; MCP: Microbial protein; CON: *n* = 3; LL, ML, and HL: *n* = 5. ^3^ T: Treat; L: Linear effect; Q: Quadratic effect.

**Table 3:** Effect of different trace element levels on VFAs levels in the rumen of growing yaks ^1^.

Item ^2^	CON	LL	ML	HL	*p*-Value ^3^		
					T	L	Q
Acetate (mmol/L)	29.44 ± 1.08	42.20 ± 8.59	45.18 ± 4.40	41.12 ± 13.32	0.12	0.07	0.06
Propionate (mmol/L)	8.35 ± 2.59	12.13 ± 4.17	15.17 ± 2.04	13.63 ± 6.87	0.22	0.08	0.22
Isobutyrate (mmol/L)	0.37 ± 0.15	0.65 ± 0.25	0.96 ± 0.19	0.77 ± 0.54	0.14	0.06	0.16
Butyrate (mmol/L)	5.22 ± 1.51 ^a^	7.48 ± 2.74 ^a^	11.82 ± 2.40 ^b^	8.55 ± 4.05 ^ab^	0.03	0.04	0.06
Isovalerate (mmol/L)	0.74 ± 0.28	1.35 ± 0.57	1.66 ± 0.56	1.64 ± 1.04	0.27	0.07	0.34
Valerate (mmol/L)	0.14 ± 0.13 ^b^	0.32 ± 0.19 ^ab^	0.58 ± 0.08 ^a^	0.47 ± 0.34 ^a^	0.05	0.02	0.17
Total VFAs (mmol/L)	44.24 ± 5.73	64.15 ± 15.72	75.57 ± 9.44	66.18 ± 25.29	0.11	0.05	0.08
Acetate/ Propionate	3.68 ± 1.01	3.72 ± 1.09	2.99 ± 0.18	3.38 ± 1.33	0.61	0.45	0.69

^1^ For each superscript, different letters indicate significant difference (*p* < 0.05), while the same letter or no letter indicates no significant difference (*p* > 0.05). Data are shown as mean ± SD. ^2^ VFAs: Volatile fatty acids; CON: *n* = 3; LL, ML, and HL: *n* = 5. ^3^ T: Treat; L: Linear effect; Q: Quadratic effect.

**Table 4:** Effect of different trace element levels on energy indexes in the serum of growing yaks ^1^.

Item	Period	CON	LL	ML	HL	*p*-Value ^2^		
						T	L	Q
Total cholesterol (mmol/L)	Before	1.74 ± 0.33	2.11 ± 0.36	2.10 ± 0.38	1.87 ± 0.26	0.35	0.62	0.08
	After	2.15 ± 0.29	2.17 ± 0.42	2.43 ± 0.30	1.87 ± 0.14	0.23	0.45	0.10
Triglyceride (mmol/L)	Before	0.30 ± 0.09 ^aA^	0.39 ± 0.10 ^aA^	0.35 ± 0.08 ^aA^	0.34 ± 0.12 ^aA^	0.67	0.72	0.31
	After	0.29 ± 0.04 ^aA^	0.40 ± 0.04 ^aA^	0.54 ± 0.11 ^bB^	0.40 ± 0.08 ^aA^	<0.01	0.01	<0.01
Glucose (mmol/L)	Before	5.37 ± 0.53	5.31 ± 0.77	5.18 ± 0.56	5.28 ± 0.46	0.98	0.78	0.79
	After	4.29 ± 0.48	4.60 ± 0.28	4.18 ± 0.60	4.46 ± 0.42	0.54	0.94	0.92

^1^ For each superscript, within the same row, values with different lowercases indicate a significant difference (*p* < 0.05), while values with the same lowercases or without lowercases indicate no significant difference (*p* > 0.05). Within the same column, values with different capital letters indicate a significant difference (*p* < 0.05), while values with the same capital letters or without capital letters indicate no significant difference (*p* > 0.05) before and after the 30-day treatment. Data are shown as mean ± SD. ^2^ T: Treat; L: Linear effect; Q: Quadratic effect; CON: *n* = 3; LL, ML, and HL: *n* = 5.

**Table 5:** Effect of different trace element levels on protein indexes in the serum of growing yaks ^1^.

Item	Period	CON	LL	ML	HL	*p*-Value ^2^		
						T	L	Q
Total protein (g/L)	Before	67.30 ± 1.80	68.20 ± 5.00	67.80 ± 2.70	68.80 ± 2.80	0.94	0.63	0.98
	After	66.50 ± 3.90	62.90 ± 2.20	64.20 ± 4.70	65.80 ± 2.60	0.43	0.90	0.08
Albumin (g/L)	Before	38.70 ± 1.80	39.90 ± 0.80	40.00 ± 1.40	40.10 ± 1.90	0.58	0.21	0.47
	After	39.80 ± 3.10	37.60 ± 2.30	38.70 ± 1.40	38.50 ± 1.10	0.58	0.53	0.28
Globulin (g/L)	Before	28.60 ± 1.20	28.30 ± 4.70	27.80 ± 2.50	28.60 ± 3.70	0.98	0.94	0.74
	After	26.70 ± 0.80	25.20 ± 1.70	25.50 ± 4.30	27.20 ± 2.60	0.65	0.71	0.17
Albumin/Globulin	Before	1.35 ± 0.10	1.43 ± 0.20	1.45 ± 0.16	1.42 ± 0.23	0.90	0.59	0.55
	After	1.49 ± 0.08	1.50 ± 0.16	1.50 ± 0.30	1.40 ± 0.10	0.81	0.67	0.38
Blood urea nitrogen (mmol/L)	Before	4.50 ± 0.82 ^aA^	4.14 ± 0.54 ^aA^	5.21 ± 0.97 ^aA^	4.48 ± 0.76 ^aA^	0.22	0.58	0.63
	After	2.15 ± 0.75 ^cB^	3.82 ± 0.32 ^abA^	4.80 ± 1.00 ^aA^	2.90 ± 1.10 ^bcB^	0.01	0.06	<0.01

^1^ For each superscript, within the same row, values with different lowercases indicate a significant difference (*p* < 0.05), while values with the same lowercases or without lowercases indicate no significant difference (*p* > 0.05). Within the same column, values with different capital letters indicate a significant difference (*p* < 0.05), while values with the same capital letters or without capital letters indicate no significant difference (*p* > 0.05) between before and after the 30-day treatment. Data are shown as mean ± SD. ^2^ T: Treat; L: Linear effect; Q: Quadratic effect; CON: *n* = 3; LL, ML, and HL: *n* = 5.

**Table 6:** Effect of different trace element levels on mineral indexes in the serum of growing yaks ^1^.

Item	Period	CON	LL	ML	HL	*p*-Value ^2^		
						T	L	Q
Potassium (mmol/L)	Before	5.56 ± 0.56	4.98 ± 0.33	5.05 ± 0.419	5.26 ± 0.26	0.25	0.37	0.06
	After	5.71 ± 0.16	5.70 ± 0.54	6.13 ± 1.24	5.56 ± 0.55	0.74	0.97	0.42
Sodium (mmol/L)	Before	137.00 ± 2.00	136.00 ± 4.00	137.00 ± 2.00	142.00 ± 6.00	0.13	0.08	0.19
	After	143.00 ± 2.00	140.00 ± 4.00	131.00 ± 19.00	139.00 ± 3.00	0.22	0.36	0.28
Chlorine (mmol/L)	Before	93.50 ± 0.90	91.66 ± 6.27	93.50 ± 0.91	98.30 ± 5.75	0.18	0.14	0.16
	After	93.70 ± 1.40	94.00 ± 3.10	87.80 ± 13.50	94.40 ± 3.90	0.54	0.80	0.35
Calcium (mmol/L)	Before	2.43 ± 0.12	2.46 ± 0.11	2.45 ± 0.07	2.41 ± 0.12	0.89	0.83	0.49
	After	2.44 ± 0.10	2.37 ± 0.07	2.20 ± 0.28	2.24 ± 0.09	0.19	0.04	0.44
Phosphorus (mmol/L)	Before	3.51 ± 0.82	2.84 ± 0.41	3.77 ± 2.33	3.14 ± 0.15	0.71	0.96	0.98
	After	3.03 ± 0.09	2.51 ± 0.76	2.94 ± 0.44	2.89 ± 0.22	0.47	1.00	0.32
Magnesium (mmol/L)	Before	0.97 ± 0.06	0.95 ± 0.07	0.96 ± 0.04	1.07 ± 0.17	0.28	0.22	0.20
	After	0.98 ± 0.04	0.93 ± 0.10	1.08 ± 0.45	1.01 ± 0.11	0.83	0.63	0.91
Iron (mmol/L)	Before	38.82 ± 10.92	37.08 ± 7.29	33.53 ± 3.68	35.58 ± 6.61	0.38	0.41	0.58
	After	30.55 ± 9.93	36.84 ± 3.96	36.15 ± 3.28	30.44 ± 7.73	0.34	0.94	0.05

^1^ For each superscript, within the same row, values without lowercases indicate no significant difference (*p* > 0.05). Within the same column, values without capital letters indicate no significant difference (*p* > 0.05) before and after the 30-day treatment. Data are shown as mean ± SD. ^2^ T: Treat; L: Linear effect; Q: Quadratic effect; CON: *n* = 3; LL, ML, and HL: *n* = 5.

**Table 7:** Effect of different trace element levels on enzyme activity indexes in the serum of growing yaks ^1^.

Item ^2^	Period	CON	LL	ML	HL	*p*-Value ^3^		
						T	L	Q
ALT/AST	Before	1.65 ± 0.18	1.77 ± 0.20	2.07 ± 0.56	1.68 ± 0.20	0.28	0.63	0.15
	After	1.37 ± 0.06	1.48 ± 0.20	1.51 ± 0.22	1.43 ± 0.29	0.85	0.67	0.35
ALP (U/L)	Before	154.00 ± 10.00	145.00 ± 30.00	125.00 ± 8.00	165.00 ± 49.00	0.28	0.85	0.13
	After	150.00 ± 28.00	155.00 ± 21.00	126.00 ± 21.00	168.00 ± 42.00	0.28	0.70	0.19
LDH (IU/L)	Before	978.00 ± 89.00	1004.00 ± 60.00	923.00 ± 166.00	952.00 ± 13.00	0.73	0.56	0.97
	After	920.00 ± 108.00	945.00 ± 112.00	848.00 ± 126.00	918.00 ± 126.00	0.67	0.69	0.69
CLP (IU/L)	Before	59.32 ± 3.63 ^aA^	68.34 ± 17.15 ^aA^	60.16 ± 14.45 ^aA^	62.35 ± 13.85 ^aA^	0.77	0.98	0.63
	After	59.65 ± 4.19 ^cA^	71.90 ± 8.81 ^aA^	81.63 ± 5.08 ^bB^	82.41 ± 2.84 ^bB^	<0.01	<0.01	0.06
T-SOD (IU/L)	Before	146.03 ± 6.23 ^aA^	151.46 ± 10.38 ^aA^	145.60 ± 10.11 ^aA^	147.07 ± 18.92 ^aA^	0.89	0.93	0.76
	After	149.28 ± 9.79 ^cA^	160.54 ± 6.54 ^aA^	172.78 ± 5.24 ^bB^	174.90 ± 6.54 ^bB^	<0.01	<0.01	0.19

^1^ For each superscript, within the same row, values with different lowercases indicate a significant difference (*p* < 0.05), while values with the same lowercases or without lowercases indicate no significant difference (*p* > 0.05). Within the same column, values with different capital letters indicate a significant difference (*p* < 0.05), while values with the same capital letters or without capital letters indicate no significant difference (*p* > 0.05) before and after the 30-day treatment. Data are shown as mean ± SD. ^2^ ALT: Glutathione aminotransferase; AST: Glutathione transaminase; ALP: Alkaline phosphatase; LDH: Lactate dehydrogenase; CLP: Ceruloplasmin; T-SOD: Total superoxide dismutase; CON: *n* = 3; LL, ML, and HL: *n* = 5. ^3^ T: Treat; L: Linear effect; Q: Quadratic effect.

**Table 8:** Comparison of the requirement of copper, manganese, and iodine for growing yaks with different nutritional standards for growing cattle.

Trace Element	This Research	NRC (1989)	NRC (2000)	ARC (1980)	NY/T (2004)	CSIRO (2007)	NorFor (2011)	Tolerance Level
Copper (mg/kg)	15.00	10.00	12.00~15.20	11.80~15.40	10.00	12.80	10.00	100.00
Manganese (mg/kg)	50.00	40.00	22.00~24.20	10.00~25.00	20.00	35.00~120.00	20.00	1000.00
Iodine (mg/kg)	0.50	——	0.25~0.50	0.50	0.50	——	0.50	50.00

## Data Availability

Data can be accessed in the article.
